# Transparency and tunable slow and fast light in a nonlinear optomechanical cavity

**DOI:** 10.1038/srep35090

**Published:** 2016-10-11

**Authors:** Ling Li, Wenjie Nie, Aixi Chen

**Affiliations:** 1 Department of Applied Physics, East China Jiaotong University, Nanchang, 330013, China; 2 Institute for Quantum Computing, University of Waterloo, Ontario N2L 3G1, Canada

## Abstract

We investigate theoretically the optical response of the output field and the tunable slow and fast light in a nonlinear optomechanical cavity with a degenerate optical parametric amplifier (OPA) and a higher order excited atomic ensemble. Studies show that the higher-order-excitation atom which is similar to the degenerate OPA that acts as a nonlinear medium, induces an additional dip in absorption spectrum of the probe field. The coherence of the mechanical oscillator leads to split the peak in absorption in the probe field spectrum so that the phenomenon of optomechanically induced transparency (OMIT) is generated from the output probe field. In particular, the presence of nonlinearities with the degenerate OPA and the higher order excited atoms can affect significantly the width of the transparency windows, providing an additional flexibility for controlling optical properties. Furthermore, in the presence of the degenerate OPA, the optical-response properties for the probe field become phase-sensitive so that a tunable switch from slow to fast light can be realized.

It is well-known that a three-level atomic medium driven by a strong controlling field can become transparent for a weak probe field, which results from the quantum interference between the two different pathways of excitation in atomic system. This phenomenon is a typical quantum coherent effect called the electromagnetically induced transparency (EIT)^1^,^2^,^3^, which has been widely investigated both in theory^4^,^5^,^6^ and in experiment^2^,^7^. It is shown that EIT is important for various applications^8^,^9^ such as slow light, light storage and the production of a giant nonlinear effect. The phenomenon of EIT has been recently observed in the other solid state systems, i.e., quantum wells^10^, metamaterial^11^ and nitrogen-vacancy centers^12^. In addition, the double EIT windows in multi-level atomic systems have been studied in detail^13^.

On the other hand, the propagation of a weak probe field in optomechanical system^14^ can be coherently manipulated through the driving of a strong coupling field which leads to an optomechanical coupling between the cavity mode and the mechanical oscillator. Several important quantum optomechanical characteristics, i.e., optomechanical entanglement^15^,^16^ and optically cooling mechanical mode^17^,^18^,^19^,^20^ as well as transitions between classical and quantum behaviors of a mechanical system^21^,^22^ have been extensively investigated by pumping the cavity with external laser fields. In particular, a phenomenon of EIT-like, called generally the optomechanically induced transparency (OMIT), has been shown theoretically^23^,^24^,^25^,^26^,^27^,^28^,^29^ and observed experimentally in optical cavities^30^,^31^,^32^,^33^ and microwave cavities^34^. OMIT can be designed to slow and switch a probe signal^35^ and further used to store light^36^. The transparency behavior in an optomechanical system also advance the ground-state cooling of mechanical motions and optomechanical entanglement between the optical and mechanical modes^37^,^38^,^39^.

Upon combining the optomechanical system with cavity quantum electrodynamics (QED)^40^, the influence of an additional atomic medium on the OMIT in a hybrid optomechanical system^41^,^42^ has been investigated in detail, where the low-atomic excitation limit of atoms requires the single-atom excitation probability to be much less than 1^43^,^44^. However, this may restrict the amplitude of the driving field in this kind of hybrid optomechanical system so that the optomechanical coupling between the optical and mechanical modes can not be advanced further by increasing the pump power. In order to relax the constraints on the driving of the system or the atom-field coupling strength, we need to go beyond the low-atomic excitation limit for the atomic medium embedded in an optomechanical system. In previous paper, we have shown that when the low excitation condition of atoms breaks slightly, a large driving but a relatively small atom-field detuning can be applied to help observe OMIT behavior in a levitated optomechanical system^45^.

In addition, we know the nonlinear optical effect of the optomechanical system can be obviously enhanced by adding a degenerate optical parametric amplifier (OPA). The degenerate OPA placed in the optomechanical system can increase the effective optomechanical coupling between the movable mirror and the cavity field, which results from increase of the photon numbers in the cavity. Some researches about the influences of the degenerate OPA on the propagation of the probe field are reported in refs 28,46,47, where the optical properties of the output field, i.e., the width of OMIT, can be controlled easily by adjusting the pump amplitude of the degenerate OPA. On the other hand, when the degenerate OPA is included, the quantum interference effect between the probe field and the generated anti-Stokes field depends strongly on the phase of the degenerate OPA so that the optical-response properties for the probe field will become phase-sensitive. As so far, the response of nonlinear optomechanical cavity with an excited atomic ensemble to a weak probe light is not reported under the condition of the existence of the degenerate OPA. In this paper, we investigate the properties of absorption and dispersion of the probe field propagating the optomechanical system including low- or high-excitation atomic medium and a degenerate OPA. Also, we contrast the role of the nonlinearities of the higher-order excitation of atoms with one of the degenerate OPA in the optical properties of the output field. Further, OMIT behavior in absorption of the probe field generated through the optomechanical coupling is discussed in detail, which is influenced by the higher order excitation of the atoms and the degenerate OPA. We also discuss in detail how to control the switch from slow to fast light of the output probe field by adjusting the phase of the field driving the degenerate OPA. The result suggests that the phase-sensitive interference effects have potential applications to provide new tools for controlling and engineering light propagation.

## Optomechanical Model and Hamiltonian

**Figure 1:**
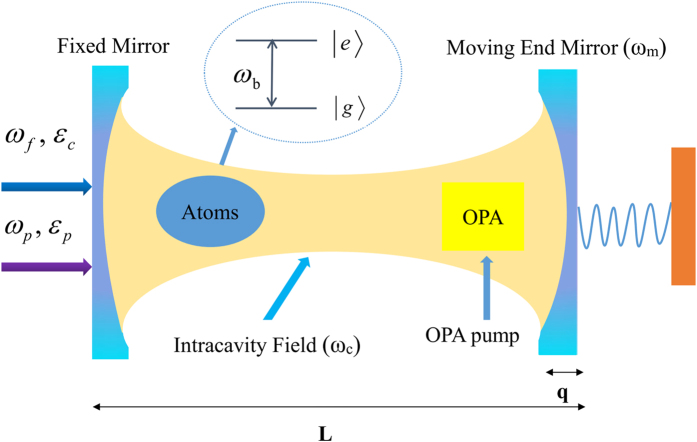
Schematic of the setup studied in the paper. The optomechanical cavity contains *N* identical two-level atoms and a degenerate OPA. The cavity mode is driven by a strong input laser field and a weak probe field through the left cavity mirror. The nonlinear crystal is pumped by an additional laser beam to produce parametric amplification.

As shown schematically in Fig. 1, the model that we consider is a nonlinear optomechanical system, where a degenerate OPA and *N* identical two-level atoms (with transition frequency *ω*_*b*_ and decay rate *γ*_*b*_) are placed in a Fabry-Férot cavity with length *L* consisting of one fixed mirror and one movable mirror. Specifically, we consider a quadrupole transition between the 6*s*^2 1^*S*_0_ ground state |*g*〉 and the 6*s*6*p*^3^*P*_1_ excited state |*e*〉 of atomic barium for the two-level atoms^48^,^49^. The transition wavelength between the two states in atomic barium and the decay rate of the excited state to the ground state are *λ* = 791 nm and *γ*_*b*_ = 47 kHz, respectively^48^. Further, we consider a realistic optical cavity, where the intracavity photon leakage can be occurred through the input and the movable mirror^33^,^50^,^51^. We assume the decay rates of two cavity mirrors are equal without loss of generality, i.e., *κ*_0_ = *κ*_1_, where *κ*_0_ and *κ*_1_ are the decay rates of the cavity field through the input and the movable mirrors, respectively.

The cavity mode with frequency *ω*_*c*_ is driven by a classical control field with frequency *ω*_*f*_ and amplitude *ε*_*c*_ as well as a weak probe field with frequency *ω*_*p*_ and amplitude *ε*_*p*_, which then exerts an optical radiation pressure on the movable cavity mirror. Moreover, the system is pumped by an additional laser beam with coupling coefficient *β* to produce parametric amplification^28^,^46^,^47^. In general, the movable mirror is treated as a quantum-mechanical harmonic oscillator with resonance frequency *ω*_*m*_, effective mass *m* and damping rate *γ*_*m*_. Then, the total Hamiltonian of the system can be written as^15^,^28^,^43^


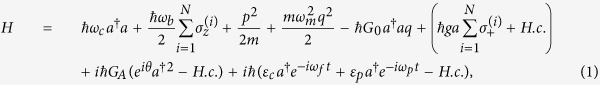


where the first term describes the free Hamiltonian of cavity field and *a (a*^†^) is the annihilation (creation) operator of the cavity mode satisfying the commutation relation [*a*, *a*^†^] = 1. The second term is the free Hamiltonian of the atomic ensemble, where the ground state and the excited state of the *i*th two-level atom are described by |*g*〉^(*i*)^ and |*e*〉^(*i*)^ and therefore 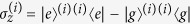. In addition, the pseudospin-1/2 operators 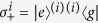 and 
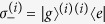
 for the *i*th atom satisfy the commutation relations 
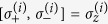
 and 
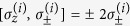
. The third and fourth terms are the kinetic and potential energies of the movable mirror, respectively; *q* and *p* are the position and momentum operators for the movable mirror with the commutation relation [*q*, *p*] = *iħ*. The fifth term describes the optomechanical coupling of the cavity mode with the movable mirror; *G*_0_ = *ω*_*c*_/*L* is the optomechanical coupling strength between the mechanical mode and cavity mode^53^. *L* is the length of the cavity. The last term in the first line denotes the interaction of the atomic ensemble with the driven cavity field, where *g* represents the averaged atom-field coupling strength^43^,^44^,^52^. The first term in the second line describes the coupling of the cavity mode with the degenerate OPA; *G*_*A*_ is the nonlinear gain of the degenerate OPA, which is proportional to the pump amplitude, *E*_*OPA*_, i.e., *G*_*A*_ = *β*|*E*_*OPA*_|; *θ* is the phase of the field driving the OPA^28^,^46^,^47^. The last terms describe the interaction of the cavity field with the coupling field and that of the cavity field with the probe field, with the amplitude 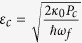 and 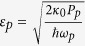, respectively. *P*_*c*_ and *P*_*p*_ are the laser powers.

For simplification, we use the Holstein-Primakoff transformation^54^, which is defined as 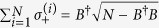, 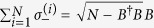 and 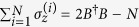, to rewrite Hamiltonian (0), where *B* and *B*^†^ satisfy the fundamental commutation relation [*B*, *B*^†^] = 1^54^. In general, in the low-excitation limit 〈*B*^†^*B*〉/*N* ≪ 1 for the atoms, the Holstein-Primakoff transformation becomes 
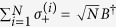
 and 
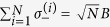
. In the present system, we assume that the low-excitation condition is broken slightly for the atomic ensemble so that the higher orders of the Holstein-Primakoff transformation should be included^45^,^55^, i.e., 
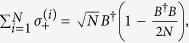
 
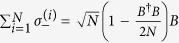
 and 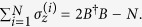 Then, in the interaction picture with respect to *H*_0_ = *ħω*_*f*_ (*a*^†^*a* + *B*^†^*B*), the Hamiltonian of the total system, Eq. (0), is rewritten as


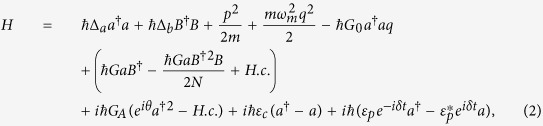


where 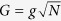 is the collective coupling strength of the atomic ensemble with the cavity field. Δ_*a*_ = *ω*_*c*_ − *ω*_*f*_, Δ_*b*_ = *ω*_*b*_ − *ω*_*f*_ and *δ* = *ω*_*p*_ − *ω*_*f*_ are the detunings. In the derivation of Eq. (2), a constant term *Nω*_*a*_/2 has been neglected.

## System Dynamics and Equation of Motion

For a detailed analysis of the system, we consider photon losses in the cavity through the input mirror with decay rate *κ*_0_ and the movable mirror with decay rate *κ*_1_; and Brownian noise acting on the mirror as well as decays associated with the atoms. Based on the Hamiltonian in Eq. (2), the quantum dynamics of the system can be described by the following Heisenberg-Langevin equation^33^:


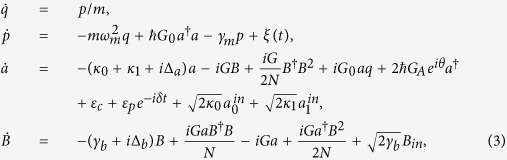


where 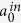, 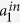 and *B*_*in*_ are the input vacuum noise operators with zero mean value corresponding to the input mirror, the movable mirror and the atomic ensemble, which are fully characterized by the nonzero correlation functions 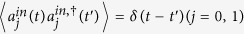 and 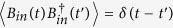, respectively^56^. *ξ*(*t*) is the Brownian stochastic force with zero mean value and its correlation function is characterized by 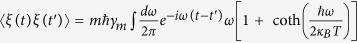
57, where *k*_*B*_ is the Boltzmann constant and *T* is the temperature of the reservoir related to the movable mirror.

In order to studying the effect of the higher order excited atomic ensemble and the degenerate OPA on the optical properties of the output field in the optomechanical system, we need to investigate the motional equations of the quantum fluctuations around the mean values. Further, the mean values at steady state for the movable mirror, atomic ensemble and cavity field can be obtained from Eq. (3) by setting all time derivatives to 0. These are found to be


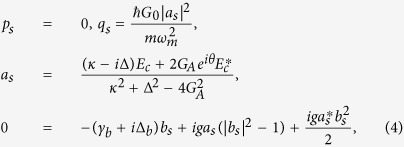


where Δ = Δ_*a*_ − *G*_0_*q*_*s*_, and 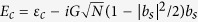 with 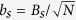. *κ* = *κ*_0_ + *κ*_1_ is the total cavity decay rate. It is seen from Eq. (4) that the steady-state values of the system depend strongly on the nonlinear gain *G*_*A*_ and the phase *θ*. Thus, the combine of the nonlinear optics and optomechanics may be used to control the optical properties of the output field.

To this end, one can split each operator in Eq. (3) into the steady-state mean value at the fixed point and a small quantum fluctuation, i.e., *O* = *O*_*s*_ + *δO*(*O* = *a*, *B*, *p*, *q*). Further, we consider that there are a large number of photons in the cavity, i.e., |*a*_*s*_| ≫ 1, so that all the higher terms (*δoδo*) in Eq. (3) can be neglected. Then, the quantum Langevin equations for the fluctuations can be written as


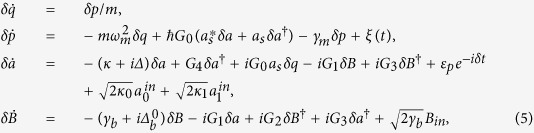


where 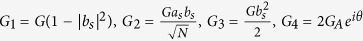 and 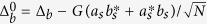. In order to solve Eq. (5), we use the ansatz *δO* = *O*_+_*e*^−*iδt*^ + *O*_−_*e*^*iδt*^. Substituting this ansatz into Eq. (5), we can obtain the following solution of interest for the response of the cavity:


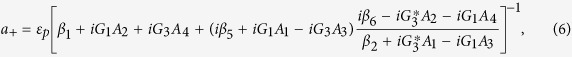


where 
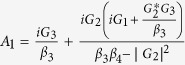
, 
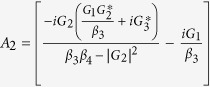
, 
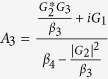
, 
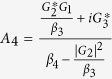
, 
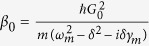
, *β*_1_ = *κ* + *i*(Δ − *δ*) − *β*_0_|*α*_*s*_|^2^, *β*_2_ = *κ* − *i*(Δ + *δ*) + *β*_0_|*α*_*s*_|^2^, 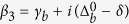, 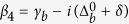, 
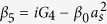
 and 
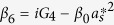
.

The expression (6) and the steady-state values in Eq. (4) help us investigate the component of the output field oscillating at the probe frequency *ω*_*p*_, which relates to the higher order excitation of the atomic ensemble |*b*_*s*_|^2^ as well as the degenerate OPA. In addition, the expression of *a*_−_ is not necessary since this describes the four-wave mixing with frequency *ω*_*p*_ − 2*ω*_*f*_ for the driving field and the weak probe field. We can calculate the response of the system to all frequencies detected by the output field via the standard input-output theory^58^ 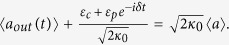 Using the above input-output relation and the ansatz *δa* = *a*_+_*e*^−*iδt*^ + *a*_−_*e*^*iδt*^, we can express the mean value of the output field as


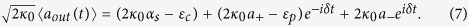


It is noted that the second term on the right-hand side of Eq. (7) corresponds to the response of the whole system to the weak probe field at frequency *ω*_*p*_. Thus, we can examine the total output field at the frequency *ω*_*p*_ by defining an amplitude of the rescaled output field corresponding to the weak probe field as


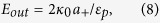


where we have removed the constant term. The real and imaginary parts of the output probe field account for in-phase and out-phase quadratures of the output field spectra and can be written as 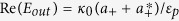 and 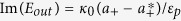, which describe the absorption and dispersion of the whole system to the weak probe field, respectively. In general, the modification of the probe response and the phenomenon of the transparency can be generated by the coupling the atoms or the mechanical motion with cavity field^41^,^42^. In the present optomechanical system, we investigate in detail the generation of dips in absorption induced by the atom-field and optomechanical couplings as well as the degenerate OPA.

Moreover, in the region of the narrow transparency window the rapid phase dispersion *φ*(*ω*_*p*_) = arg(*E*_*out*_) can cause the group delay expressed as 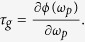 A positive group delay with *τ*_*g*_ > 0 corresponds to slow light propagation and a negative group delay with *τ*_*g*_ < 0 corresponds to fast light propagation^30^,^32^,^59^. In the following section, we also investigate theoretically a tunable switch from slow to fast light in the nonlinear optomechanical cavity by adjusting the nonlinear gain of the OPA and the phase of the field driving the OPA.

## Results

In this section, we numerically evaluate the values of phase quadratures Re(*E*_*out*_) and Im(*E*_*out*_) and quantify the slow and fast light effects through the corresponding output field *a*_+_. Further, in order to demonstrate the phenomenon of transparency and the group delay of the probe field in the system, we select the accessible parameters in optomechanical systems^43^,^60^, i.e., the wavelength of the driving field *λ*_*f*_ ≈ 791 nm, the total cavity length *L* = 0.001 m, the total cavity decay rate *κ* = 2*π* × 215 × 10^3^ Hz and *κ*_0_/*κ* = 0.5, the frequency of the moving mirror *ω*_*m*_ = 2*π* × 947 × 10^3^ Hz, the mechanical factor *Q* = *ω*_*m*_/*γ*_*m*_ = 6700, and the mass of the oscillating mirror *m* = 25 ng. In addition, we choose the parameters of atoms, i.e., the number of the atoms *N* = 10^6^, the atom-field coupling strength *g* = 2*π* × 2.3 × 10^2^ Hz and the decay rate of atom *γ*_*b*_ = 2*π* × 7.5 × 10^3^ ≈ 47000 Hz^43^,^48^. We also consider that the cavity is driven on its red sideband, i.e., Δ = *ω*_*m*_. The atom-field detuning Δ_*b*_ and the driving strength of the optical cavity *ε*_*c*_ can be calculated in terms of the excitation number of the atoms |*b*_*s*_|^2^ and the steady-state value *b*_*s*_.

### Transparency effect

We first consider that the low-excitation condition of atoms is satisfied, i.e., |*b*_*s*_|^2^ ≪ 1. Moreover, the degenerate OPA and the optomechanical coupling between the cavity field and the movable mirror are also removed or turned off, i.e., *G*_*A*_ = 0 and *G*_0_ = 0. In this case, the expression of *a*_+_ in Eq. (6) can be simplified as


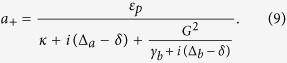


**Figure 2:**
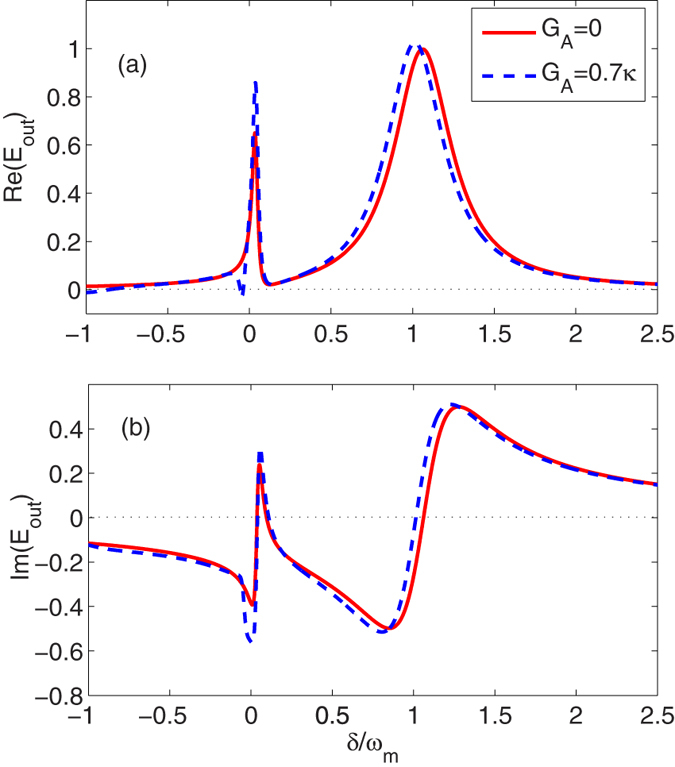
The absorption Re(*E*_*out*_) (**a**) and dispersion Im(*E*_*out*_) (**b**) are plotted as a function of *δ*/*ω*_*m*_ in the absence and presence of the degenerate OPA. Here the optomechanical coupling is turned off and the low-excitation limit for the atoms is satisfied with *b*_*s*_ = −0.01 + 0.04*i*. When the degenerate OPA is included, we select the parameters *G*_*A*_ = 0.7*κ* and *θ* = 3*π*/2. Other parameters are chosen to be *λ*_*f*_ = 791 nm, *L* = 0.001 m, *κ* = 2*π* × 215 × 10^3^ Hz, Δ = Δ_*a*_ = *ω*_*m*_ = 2*π* × 947 × 10^3^ Hz, *N* = 10^6^, *g* = 2*π* × 2.3 × 10^2^ Hz and *γ*_*b*_ = 2*π* × 7.5 × 10^3^ Hz.

It is found that the denominator of the response function is quadratic in *δ* so that the coherent coupling between the atomic collective mode and the optical mode can lead to a dip in absorption obtained as 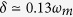 and therefore the generation of the transparency behavior which is shown in Fig. 2(a), where we show the absorption Re(*E*_*out*_) of the output field as a function of *δ*/*ω*_*m*_ with *b*_*s*_ = −0.01 + 0.04*i*; the corresponding driving strength *ε*_*c*_ = 35.12*κ*, atom-field detuning Δ_*b*_ = 0.43*κ* and excitation number of atoms |*b*_*s*_|^2^ ≈ 0.002. We can see from Fig. 2(a) that the positions of two peaks in absorption appear at 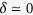 and 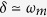, where the left peak in absorption results from the atom-field coupling^42^.

In order to compare with the above situation, we now explore the effect of the degenerate OPA on the atom-field coupled system. Correspondingly, the expression of the response of *a*_+ _ in Eq. (6) is simplified as


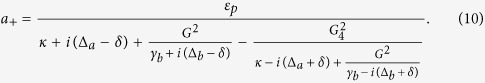


The denominator of the response function is quadruplicate in *δ*. Then, under the condition of certain parameters, we can obtain more dips in absorption. Indeed, when the degenerate OPA is included in the system, i.e., *G*_*A*_ = 0.7*κ* and *θ* = 3*π*/2 in Fig. 2(a), an additional dip in absorption appears near the left peak in absorption, i.e., 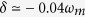, which results from the coupling between the degenerate OPA and the cavity field. Thus, we can obtain two transparent windows. Figure 2(b) depicts the dispersion shapes of the output field in the absence and presence of the degenerate OPA.

The additional dip in absorption that is similar to the one induced by the degenerate OPA can be generated by the higher order excitation of the atomic ensemble. In this case of presence of the higher order excited atomic ensemble and absence of the degenerate OPA and the optomechanical coupling, the amplitude of the output field [Eq. (6)] can be simplified as


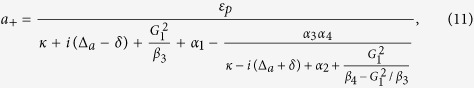


where 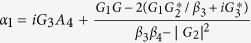, 
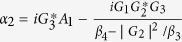
, *α*_3_ = *G*_1_*A*_1_ − *G*_3_*A*_3_ and 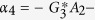  *G*_1_*A*_4_ are the terms related to the higher order of the Holstein-Primakoff transformation. One can clearly observe from Eq. (10) and Eq. (11) that the higher order coupling terms of the atoms play a role of nonlinear medium similar to the degenerate OPA for the generation of the transparency behavior.

**Figure 3:**
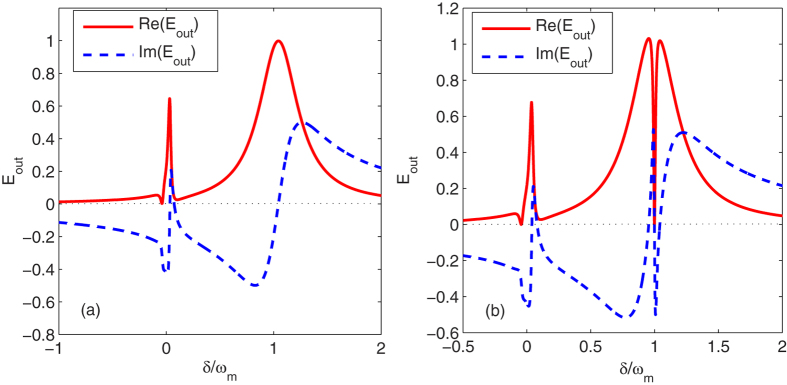
The absorption Re(*E*_*out*_) and dispersion Im(*E*_*out*_) are plotted as a function of *δ*/*ω*_*m*_ with *b*_*s*_ = −0.25 + 0.40*i*. The frequency of the moving mirror *ω*_*m*_ = 2*π* × 947 × 10^3^ Hz, the mechanical factor *Q* = *ω*_*m*_/*γ*_*m*_ = 6700 and the mass of the oscillating mirror *m* = 25 ng. Other parameter values are the same as in Fig. 2. (**a**) In the absence of the degenerate OPA and the optomechanical coupling. (**b**) In the presence of the degenerate OPA and the optomechanical coupling.

In Fig. 3(a), we show the absorption Re(*E*_*out*_) and dispersion Im(*E*_*out*_) of the output field in the absence of the degenerate OPA as a function of *δ*/*ω*_*m*_, where we select *b*_*s*_ = −0.25 + 0.40*i* so that the excitation number of the atomic ensemble |*b*_*s*_|^2^ = 0.22, the driving strength of the cavity *ε*_*c*_ = 267.5*κ* and the atom-field detuning Δ_*b*_ = 0.20*κ*. We see from Fig. 3(a) that for the selected system parameters there exists indeed an additional dip in absorption at 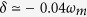 which results from the higher order excitation of the atomic ensemble, corresponding to the terms of *α*_1,2,3,4_. Further, the higher order terms related to the |*b*_*s*_|^2^ influence slightly the position of the dip in absorption at 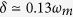 as well as the peaks in absorption; such as, the dip in absorption is attained at 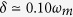. In particular, when the low-excitation condition of atoms breaks slightly, a large driving strength i.e., *ε*_*c*_ = 267.5*κ* but a relatively small atom-field detuning i.e., Δ_*b*_ = 0.20*κ* can be applied to help observe the transparency behavior in the atom-field coupled system. In contrast, the low-excitation limit of atoms requires a small driving of the cavity but a relatively large atom-field detuning. For example, in the low-excitation limit, i.e., |*b*_*s*_|^2^ = 0.002, the driving strength *ε*_*c*_ = 35.12*κ* and the atom-field detuning Δ_*b*_ = 0.43*κ* could be used [see Fig. 2].

Figure 3(b) demonstrates the transparency behavior in absorption of output probe field in the presence of the optomechanical coupling between the cavity field and the movable mirror when the degenerate OPA and the higher order excitation of the atomic ensemble are both included, i.e., *G*_*A*_ = 0.7*κ* Hz and *b*_*s*_ = −0.25 + 0.40*i*. In this case, |*b*_*s*_|^2^ = 0.22, *ε*_*c*_ = 140.78*κ* and Δ_*b*_ = 0.21*κ*. We can easily find from Fig. 3(b) that there exist three dips in absorption, where the peak in absorption at 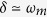 splits and therefore the optomechanically induced transparency (OMIT) appears due to the coupling between the cavity field and the movable mirror. This is because in general the strongest optomechanical coupling obtains at 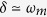 so that the probe beam interferes destructively with the anti-Stokes field generated by the mechanical oscillator and therefore a transparency window appears in the probe transmission spectrum^24^. Moreover, the positions of the left side of the two dips in absorption are 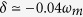 and 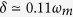, respectively, which depends on the atom-field coupling and the degenerate OPA.

**Figure 4:**
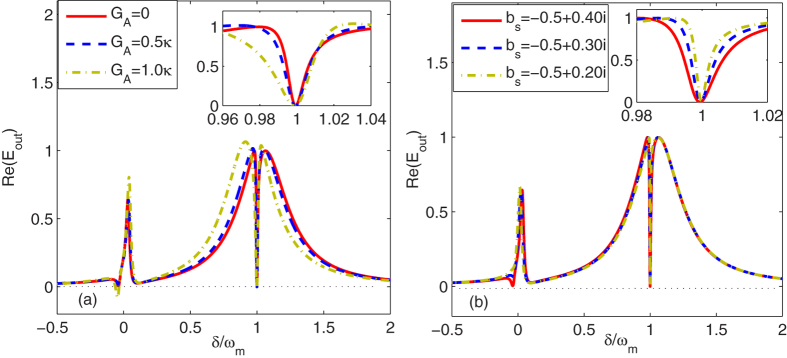
The absorption Re(*E*_*out*_) is plotted as a function of *δ*/*ω*_*m*_ with (**a**) different *G*_*A*_’s and (**b**) different *b*_*s*_’s. Other parameter values are the same as in Figs 2 and 3.

We can study the effect of the degenerate OPA on the transparency behaviors in the nonlinear optomechanical system with a higher order excited atomic medium. In Fig. 4(a), we show the absorption Re(*E*_*out*_) in the presence of the OPA as a function of *δ*/*ω*_*m*_ with different *G*_*A*_’s. Other parameter values are the same as in Figs 2 and 3. From Fig. 4(a), we see clearly that the larger the nonlinear gain of the degenerate OPA, the wider the OMIT windows. This is because the photon number in the cavity increases with increasing *G*_*A*_ [see Eq. (4)]. Therefore, the effective optomechanical coupling *G*_0_*a*_*s*_ can be increased by adding a degenerate OPA inside the optical cavity, which leads to widen the OMIT window. This suggest that the nonlinear gain of the degenerate OPA can be used to control the optical-response properties in the nonlinear optomechanical system and the width of OMIT window. Further, in the presence of both the higher order excitation of the atoms and the degenerate OPA, the left dip in absorption shallows, i.e., *G*_*A*_ = 0.5*κ* corresponds to the blue dashed line in Fig. 4(a). This means that the roles of the higher order nonlinearity of atoms and the degenerate OPA cancel each other for generating the dip in absorption in the output field. When the nonlinear gain of the OPA becomes larger, i.e., *G*_*A*_ = 1.0*κ* in Fig. 4(a), the degenerate OPA plays a determined role for the transparency of the probe field and therefore the left transparency window reappears.

We now discuss the important role of excitation of the atomic ensemble in the properties of the output field and OMIT. In Fig. 4(b), we show the absorption Re(*E*_*out*_) as a function of *δ*/*ω*_*m*_ with different *b*_*s*_’s in the absence of the degenerate OPA. We consider the three cases with *b*_*s*_ = −0.25 + 0.40*i*, *b*_*s*_ = −0.25 + 0.30*i* and *b*_*s*_ = −0.25 + 0.20*i*. The corresponding excitation numbers are calculated as |*b*_*s*_|^2^ = 0.22, |*b*_*s*_|^2^ = 0.15 and |*b*_*s*_|^2^ = 0.10, respectively. In addition, the corresponding atom-field detunings are Δ_*b*_ = 0.20*κ*, Δ_*b*_ = 0.22*κ* and Δ_*b*_ = 0.23*κ*, respectively, and driving strengths of the optical cavity are *ε*_*c*_ = 267.50*κ*, *ε*_*c*_ = 191.99*κ* and *ε*_*c*_ = 137.23*κ*, respectively. From Fig. 4(b), we see that the increase of the excitation number of the atoms widens the OMIT window. This is because when the low-excitation condition of atoms is broken slightly, the effective optomechanical coupling strength is enhanced by the increase of the photon number, which depends strongly on the higher order excitation number of atoms. In addition, the additional dip in absorption shallows so that the left transparency behavior disappears when the excitation number decreases. These results can be applied to determinate the excitation number of atomic ensemble and its important role in the properties of the output field.

### Tunable slow and fast light

**Figure 5:**
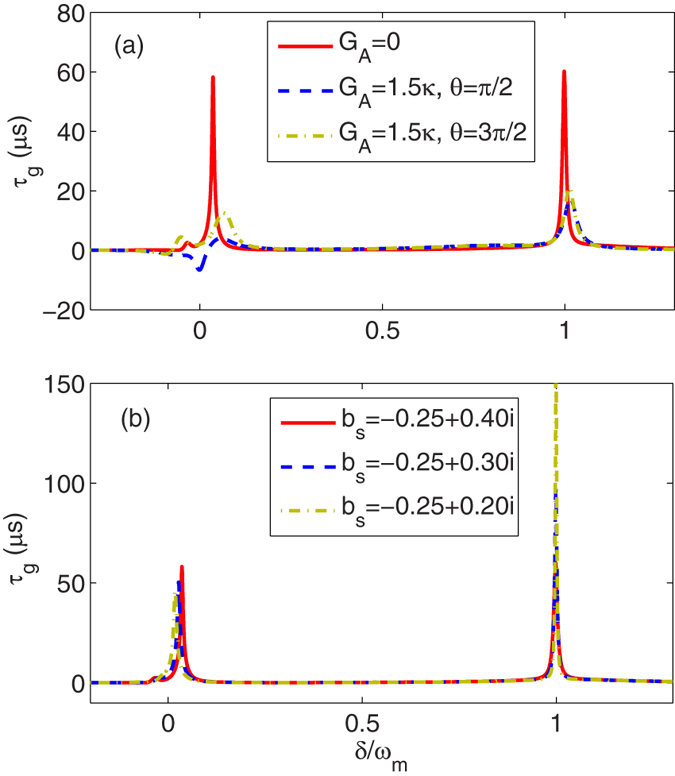
Group delay *τ*_*g*_ is plotted as a function of *δ*/*ω*_*m*_ with different *G*_*A*_’s and *θ*’s (*b*_*s*_ = −0.25 + 0.4*i*) (**a**) and different *b*_*s*_’s (*G*_*A*_ = 1.5*κ*) (**b**). Other parameter values are the same as in Fig. 4.

As mentioned in previous section, the optical response of the system to the weak probe field can be described by the group delay. In Fig. 5, the group delay of the output field at the frequency of the probe field is plotted as a function of *δ*/*ω*_*m*_ with different *G*_*A*_’s and *θ*’s (a) and different *b*_*s*_’s (b). Here we consider the driving of the cavity field is so large that the low-excitation condition of atoms is broken slightly. For example, in Fig. 5(a) we still select *b*_*s*_ = −0.25 + 0.4*i* and therefore |*b*_*s*_|^2^ = 0.22. This leads to generate an additional dip in absorption at *δ* ≈ 0 even in the absence of the degenerate OPA [see the red line in Fig. 4(b)]. In Fig. 5(b), we always remove the degenerate OPA, i.e., *G*_*A*_ = 0 and focus justly on the effect of the higher-order excitation of atoms on the optical properties of the system. Other parameter values are the same as in Fig. 4.

We see clearly from Fig. 5(a) that in the absence of the degenerate OPA, a positive group delay of the output field is obtained at *δ* ≈ 0 or *δ* ≈ *ω*_*m*_, which corresponds to the slow light effect of the output probe field and results from the enhancement of the optical transparency induced by the higher order excitation of the atoms and the optomechanical coupling, respectively [see the red line in Fig. 5(a)]. When the degenerate OPA is included in the system, i.e., *G*_*A*_ = 1.5*κ* and *θ* = *π*/2, the group delay at *δ* ≈ 0 can be turned to negative value and therefore the output probe field contains fast light. In contrast, the positive group delay at *δ* ≈ *ω*_*m*_ is decreased due to the effect of the degenerate OPA. Moreover, when the degenerate OPA with the phase *θ* = 3*π*/2 is included, the group delays at *δ* ≈ 0 and *δ* ≈ *ω*_*m*_ can be decreased due to the influence the degenerate OPA. In Fig. 5(b), we see that in the absence of the degenerate OPA, the group delay of the output field is always positive with different steady-state excitation number. Thus, only the phenomenon of slow light can be appeared.

**Figure 6:**
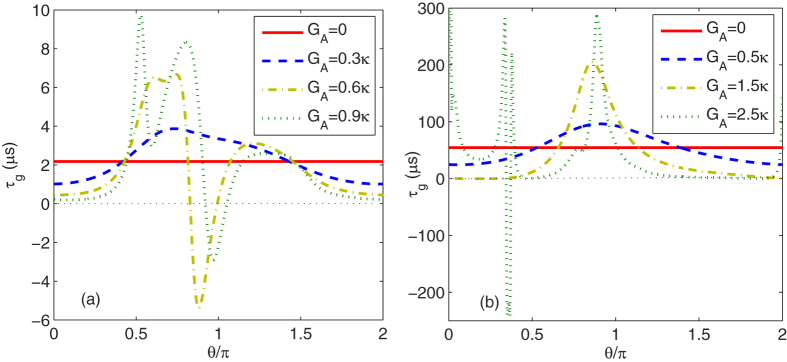
Group delays *τ*_*g*_ at (**a**) *δ* ≈ 0 and (**b**) *δ* ≈ *ω*_*m*_ are plotted as a function of the phase *θ*/*π* with different *G*_*A*_’s. Other parameter values are the same as in Fig. 4.

In Fig. 6, we show the group delays *τ*_*g*_ at *δ* ≈ 0 and *δ* ≈ *ω*_*m*_, respectively, as a function of the phase *θ*/*π* with different *G*_*A*_’s. In Fig. 6(a), we can see that in the absence of the degenerate OPA, i.e., *G*_*A*_ = 0 Hz, the group delay with *δ* ≈ 0 induced by the higher order excitation of the atoms is positive with *τ*_*g*_ = 2.2*μ*s and the transmitted probe field contains slow light. In the presence of the degenerate OPA, i.e., *G*_*A*_ = 0.3*κ*, the positive group delay is not a monotonous function of the phase and a peak appears in the intermediate value of the phase. When the nonlinear gain of the OPA becomes larger, i.e., *G*_*A*_ = 0.6*κ*, the group delay can be negative corresponding to the fast light within a finite interval of the phase around *θ* = 0.88*π*, whose position moves in the positive direction with increasing *G*_*A*_ for the selected range of the parameters [see the dash-dot and dot lines in Fig. 6(a)]. Physically, when the degenerate OPA is added inside the atom-field coupled system, the quantum interference effect between the probe field and the anti-Stokes field generated by the atoms is related directly to the phase of the degenerate OPA by the coupling coefficient *G*_4_ [see Eq. (6) or Eq. (10)]. Therefore, the optical-response properties for the probe field become phase-sensitive so that a tunable switch from slow to fast light can be realized by adjusting the phase of the degenerate OPA.

In Fig. 6(b), we can see that in the absence of the degenerate OPA, i.e., *G*_*A*_ = 0, the group delay with *δ* ≈ *ω*_*m*_ induced by the OMIT is *τ*_*g*_ = 54.5*μ*s, which is much greater than that induced by the higher order atomic excitaiton. When the degenerate OPA is included, i.e., *G*_*A*_ = 0.5*κ*, the effect of the degenerate OPA on the optomechanical system leads to the increase in the group delay for the intermediate value of the phase. In particular, when the nonlinear gain of the OPA becomes larger, i.e., *G*_*A*_ = 2.5*κ*, a negative group delay and therefore the fast light appears in a small interval of the phase around *θ* = 0.36*π*. Similarly, the tunability of the group delay induced by the OMIT results from the quantum interference effect between the probe field and the anti-Stokes field generated by the mechanical oscillator, which depends strongly on the phase of the degenerate OPA. These results suggest that the phase-sensitive interference effect can be used to control the light propagation from slow to fast light of the transmitted probe field.

## Discussion

In conclusion, we have studied a nonlinear optomechanical cavity with a degenerate OPA and a higher order excited atomic ensemble, which is driven by pump and probe laser fields. We derive the expression of the response of probe field in the system and demonstrate numerically the optical properties of the output field with experimentally accessible parameters. It is shown that there exist three dips in absorption in the output field in the presence of a higher order excited atomic medium, which induced by the optomechanical and atom-field couplings as well as the higher order excited terms related to the atom-field coupling, respectively. Further, the OMIT behavior in absorption of probe field can be generated through the optomechanical coupling between the cavity field and the movable mirror. We show that the higher order excitation of the atoms and the degenerate OPA can significantly affect the width of these transparency windows, which can be applied to determinate the excitation number of atoms and the important roles of nonlinear media in the optical properties of the output field. In particular, we find that the higher order excitation of the atoms and the degenerate OPA play a similar role of nonlinear medium in the generation of the additional transparency behavior in the probe field spectrum. We also discuss in detail the change in the magnitude of the group delay as well as a tunable switch from slow to fast light of the output probe field, where the group delay tunability is mainly due to the phase of the degenerate OPA. The phase-sensitive interference effects in the optical-response properties have potential applications to provide new tools for controlling and engineering light propagation.

## Additional Information

**How to cite this article**: Li, L. *et al.* Transparency and tunable slow and fast light in a nonlinear optomechanical cavity. *Sci. Rep.* **6**, 35090; doi: 10.1038/srep35090 (2016).
